# Trends in the use of benzodiazepine receptor agonists among working-age adults in Belgium from 2004 to 2018

**DOI:** 10.3389/fpubh.2023.1191151

**Published:** 2023-06-15

**Authors:** Lisa Colman, Katrijn Delaruelle, Piet Bracke, Melissa Ceuterick

**Affiliations:** Department of Sociology, HeDeRa (Health and Demographic Research), Ghent University, Ghent, Belgium

**Keywords:** benzodiazepines, education, employment, health inequities, medicalization, mental health, psychotropic drugs

## Abstract

**Introduction:**

The use of psychotropics, such as benzodiazepine receptor agonists (BzRAs), among working-age adults in Belgium has shown educational differences. However, it is unclear how work status plays a role in this relationship. Therefore, this research aims to investigate whether work status explains observed educational differences in BzRA use. In addition, considering medicalisation processes, where non-medical factors, such as work status, are increasingly associated with medical mental health care-seeking behavior, this research also aims to investigate whether work status explains observed educational differences in BzRA use, regardless of mental health status.

**Methods:**

Data are obtained from the Belgian Health Interview Survey (BHIS). Four successive waves are covered: 2004, 2008, 2013, and 2018. The weighted data represent a sample of 18,547 Belgian respondents aged 18 to 65 years old. Poisson regression models are used to analyze the research aims. Time evolutions are plotted using marginal means postestimation.

**Results:**

The average use of BzRAs shows a slight decline over the waves studied (2004 = 5.99, 2008 = 5.88, 2013 = 5.33, 2018 = 4.31). Educational and work status differences in BzRA use are observed, regardless of mental health status. Individuals with longer education report lower use compared to individuals with shorter education, and individuals who are unemployed, (pre-)retired, or sick or disabled report higher use compared to employed individuals. Furthermore, work status acts as a mediator, partially explaining educational differences in BzRA use, regardless of mental health status.

**Discussion:**

Work-related uncertainty leads to increased prescribing and medication use, regardless of mental health. Medicalisation and pharmaceuticalisation processes detach social problems from their social roots and treat them as personal failures. The marginalization of the social roots of unemployment, sick leave and involuntary (pre-)retirement has led to a personalization of responsibility. Negative feelings arising from such work statuses may cause isolated, non-specific symptoms for which medical treatment is sought.

## Introduction

Benzodiazepine receptor agonists (BzRAs), which include benzodiazepines and Z-products, are widely prescribed for the treatment of anxiety, nervousness and sleep problems (1–5). While BzRAs can have initial beneficial anxiolytic and sedative effects, their use also leads to risks of developing tolerance, dependence, withdrawal symptoms and severe adverse effects, such as impaired cognitive functioning and driving skills and an increased risk of falls and hip fractures (6, 7). These risks already appear after 2 to 4 weeks of use and are particularly elevated among long-term users (8).

For over two decades, the Belgian Federal Service of Public Health has launched awareness campaigns to decrease anxiolytic-sedative consumption in Belgium (9). Reports and studies that followed have mainly focused on BzRA use by older Belgians (65+ years) (10–12). Hence, it is also important to examine other understudied age groups, such as working-age adults, who are especially prone to experience daily mental stressors from multiple life domains, such as work and personal life. These stressors, along with work-life spillover effects, may influence BzRA use (13, 14).

Recent Belgian studies on the use of psychotropics, like benzodiazepines, Z-products and antidepressants, have shown clear educational differences in use among working-age adults, with these differences persisting over time (15, 16). These educational differences in use may be partially attributed to differences in work status. Education is the most important mechanism of labor market allocation in credential societies and strongly influences work status (17). However, while work status is a crucial factor to consider when analyzing BzRA use among working-age adults, no prior empirical studies, to the best of our knowledge, have examined this. Therefore, this research aims to explore the mediating role of work status in relation to BzRA use.

Mostly, researchers assume that individuals with shorter education are more at risk of using BzRAs because they are less likely to have a favorable and stable work status, with an increased risk of unemployment and involuntary (pre-)retirement (18–22), which may cause mental health complaints. Studies have already shown that unemployed individuals are more likely to experience poor mental health, and to make greater use of mental health care, including taking antidepressants (23, 24). Unemployed individuals also have a higher chance of substance abuse (25), and pre-retirement as well, relates to higher levels of mental distress (26).

However, we argue that work status may also mediate the relationship between education and the use of BzRAs independently of mental health complaints, due to medicalisation processes. According to Conrad (27) medicalisation is a social process in which normal biological processes or behaviors are increasingly described, accepted and treated as medical problems, leading to increased attention for- and growing consumption of medical treatments. Given these medicalisation processes, non-medical factors, such as work status, are increasingly associated with medical mental health care-seeking behavior (27). Work status is increasingly viewed as a personal responsibility, whereas ‘not being employed' is seen as an individually caused situation (28, 29), with all the negative mental health consequences this may entail.

The purpose of this study is to describe trends in BzRA use among working-age adults in Belgium and to assess (1) to what extent work status explains observed educational differences in BzRA use, and (2) to what extent work status explains observed educational differences in BzRA use, regardless of mental health status. We assume that work status differences in BzRA use, irrespective of mental health status, provide indications of medicalisation. To answer these research questions, data from the Belgian Health Interview Survey (BHIS) covering a wide time span (2004–2018) are used to analyze evolutions in education and work status and differences in BzRA use.

## Methods

### Sample

The Belgian Health Interview Survey (BHIS) is a repeated cross-sectional survey coordinated by Sciensano, the Scientific Institute of Public Health of the federal Belgian State. Four successive waves are covered: 2004, 2008, 2013, and 2018. Households and their members are selected from the National Register following a multi-stage stratified sampling procedure. Information is collected through face-to-face interviews, as well as through a self-administered questionnaire. After pooling the data and omitting cases with missing values, the final sample includes 18,547 Belgian respondents of working age between 18 and 65 years old (2004 = 5,045 respondents, 2008 = 4,412 respondents, 2013 = 4,076 respondents, 2018 = 5,014 respondents). The waves are well balanced in terms of gender distribution (ratio men/women: 2004 = 49.42/50.58, 2008 = 49.09/50.91, 2013 = 48.77/51.23, 2018 = 49.68/50.32). The average age of respondents across the waves is slightly older, with a mean of 44 years old (${\bar{X}}_{2004}$ = 43.40, ${\bar{X}}_{2008}$ = 43.56, ${\bar{X}}_{2013}$ = 44.51, ${\bar{X}}_{2018}$ = 45.60). Information on respondent selection criteria and data cleaning are available in Appendix 1_a_, as well as more information on general sample characteristics (see Appendix 1_b_).

### Variables

#### Dependent variable

The dependent variable ‘benzodiazepine receptor agonists (BzRA) use' measures the use of benzodiazepines and/or Z-products in the past 24 h.^1^ Respondents were asked to show the medicines that they had used in the past 24 h, and the interviewer indicated on a list whether the medicines belonged to a specific Anatomical Therapeutic Chemical (ATC) category, which is an internationally accepted classification system for medicines (30). The computed variable consists of two categories: 0 = no BzRA use in the past 24 h, 1 = BzRA use in the past 24 h.

#### Independent variable

Education is measured as the highest level of education completed and is recoded into three categories according to the International Standard Classification of Education (ISCED) of 2011 (31): 1 = shorter education (pre-primary or primary education), 2 = intermediate education (lower- and upper secondary education) and 3 = longer education (post-secondary or tertiary education) [ref.cat].

#### Mediation variable

Work status is recoded into five categories: 1 = employed [ref.cat], 2 = unemployed, 3 = non-employed (consisting of two items; housekeeping without benefits and family worker), 4 = sick or disabled and 5 = (pre-)retired. Being sick or disabled is added as a separate category due to a known association with BzRA use (32, 33).

#### Covariate variables

Several other important variables are considered: household income is recoded into four categories: 1 = low income (1^st^ and 2^nd^ quintile), 2 = medium income (3^rd^ and 4^th^ quintile), 3 = high income (5^th^ quintile) [ref.cat] and 4 = information missing. Gender (0 = man) and country of birth (0 = Belgium) are included as dichotomous variables. Age is recoded into three categories: 1 = 18-34 years old [ref.cat], 2 = 35–49 years old, 3 = 50–65 years old as well as urbanization: 1 = cities-agglomerates [ref.cat], 2=urban-suburban, 3=rural and region: 1 = Flanders [ref.cat], 2 = Brussels, 3 = Wallonia. Wave is incorporated as a categorical variable: 1 = 2004 [ref.cat], 2 = 2008, 3 = 2013, 4 = 2018. GP-contact in the past twelve months (0 = no) and regular GP (0 = no) are added as dichotomous variables along with frequency of social contact (0 = less than once a week). Household composition consists of three categories: 1 = single or one parent household [ref.cat], 2 = couple with or without children living in the household, 3 = another household composition.

This study includes three variables to account for mental health status, namely the presence of depression complains, anxiety complaints and sleeping complaints. Depression complaints (0 = no) is operationalized as a dichotomous variable based on the Patient Health Questionnaire (PHQ-9) (34), which assesses depression symptoms experienced over the past 2 weeks. Additional details on the operationalization of depression complaints are provided in Appendix 2_a_. Similarly, anxiety complaints is operationalized as a dichotomous variable (0 = no) based on the Generalized Anxiety Disorder questionnaire (GAD-7) (35), which measures anxiety symptoms over the past 2 weeks. Further information on the operationalization of anxiety complaints is available in Appendix 2_b_. Lastly, a metric variable is included to measure sleeping complaints, specifically, “loss of sleep over worry” experienced in the past 2 weeks.

### Statistical procedure

Prevalence rates of BzRA use are calculated using weighted proportions and are stratified by wave and education, as well as wave and work status. Prevalence rates of work status are also reported stratified by education, as well as prevalence rates of BzRA use, stratified by work status and education. After conducting bivariate statistics, four Poisson regression models are estimated. The models estimate incidence rate ratios (IRRs), Z-scores and their corresponding *P*-values. The first two models are tested without the mental health variables added, with one model without work status included and one model with work status included. The next two models are tested with the mental health variables added, again with one model without work status included and one model with work status included. To concentrate on the mediating effect of work status, we control for several variables that are both related to education and BzRA use (such as income, GP contact, social contact, and so on). Additionally, the results of the tested models are plotted, using marginal means postestimations. Analyses are weighted for survey sampling and non-participation bias and are conducted with SPSS 28 and STATA 15.

## Results

Bivariate results presented in Table 1 indicate that individuals with longer education (2.92%) use BzRAs less frequently compared to those with intermediate (6.22%) and shorter education (12.54%). The results also reveal that employed individuals (2.60%) use them the least, while unemployed individuals (7.48%) use them more often than non-employed individuals (6.51%). (Pre-)retired individuals (11.17%) use BzRAs more frequently than those in the previous work status categories. Notably, individuals who are sick or disabled (31.38%) use them significantly more frequently. The results also show a significant decline in use over the observed period (2004 = 5.99%; 2018 = 4.31%). The differences in use based on education and work status remain consistent across the waves. However, in terms of the education gradient, the differences in the last wave are less pronounced due to the sharp decline in use by the shorter educated.

**Table 1:** Weighted proportions^a^ of BzRA use in the past 24 h, according to wave, stratified by education and work status.

	**2004–2018**	**2004**	**2008**	**2013**	**2018**	
	**%**	**%**	**%**	**%**	**%**	
*N*	18,547	5,045	4,412	4,076	5,014	*P*-values^b^: < 0.05
	5.34	5.99	5.88	5.33	4.31	
*N*	18,547	5,045	4,412	4,076	5,014	*P*-values^b^: < 0.001
Shorter education	12.54	12.43	13.84	14.34	7.56	
Intermediate education	6.22	6.40	6.40	6.36	5.77	
Longer education	2.92	3.31	3.38	2.71	2.48	
*N*	18,547	5,045	4,412	4,076	5,014	*P*-values^b^: < 0.001
Employed	2.60	3.27	3.20	2.36	1.74	
Unemployed	7.48	7.99	8.28	5.23	8.19	
Non-employed	6.51	9.78	6.60	3.37	4.39	
Sick or disabled	31.38	29.87	34.00	35.13	27.76	
(Pre-)retired	11.17	11.57	12.10	11.71	8.90	

^a^Crude rates. unadjusted for other indicators. Weighted for survey sampling and non-participation bias. ^b^Weighted Pearson chi-square test for the entire studied period. P-values are lower than 0.05, which indicates that BzRA use significantly differs over the waves; *P*-values are lower than 0.001, which indicates that the categories of education or work status significantly differ from each other as it comes to BzRA use.

The results in Table 2 indicate that individuals with longer education (87.31%) are more likely to be employed compared to those with intermediate (67.91%) and shorter education (38.18%). Moreover, individuals with shorter education are more likely to be unemployed, non-employed, (pre-)retired, or sick or disabled than those with intermediate and longer education. Therefore, significant educational differences exist in work status. Regarding BzRA use among different work status categories stratified by education, significant educational differences are observed within the work status categories. In the employed category, individuals with longer education (1.94%) use fewer BzRAs compared to those with shorter education (5.18%), and comparable results are found for the other categories, with major differences. For example, in the non-employed category, individuals with shorter education (10.79%) use them significantly more than those with longer education (1.33%).

**Table 2:** Weighted proportions^a^ of work status, stratified by education; and weighted proportions^a^ of BzRA use in the past 24 h according to work status, stratified by education.

	**Employed %**	**Unemployed %**	**Non-employed %**	**Sick or disabled %**	**(Pre-)retired %**	
*N* (2004–2018)	13,317	1,544	1,102	923	1,661	*P*-values^b^: < 0.001
Shorter education	38.18	14.86	15.42	12.71	18.82	
Intermediate education	67.91	9.69	6.62	6.43	9.36	
Longer education	87.31	3.19	2.34	1.85	5.31	
Shorter education (*N* = 1,427)	5.18	10.34	10.79	37.29	13.91	*P*-values^b^: < 0.001
Intermediate education (*N* = 9,379)	3.05	7.17	6.55	30.79	11.18	*P*-values^b^: < 0.001
Longer education (*N* = 7,741)	1.94	6.28	1.33	26.73	9.40	*P*-values^b^: < 0.001

^a^Crude rates. unadjusted for other indicators. Weighted for survey sampling and non-participation bias. ^b^Weighted Pearson chi-square test for the entire studied period. *P*-values are lower than 0.001, which indicates that the categories of work status significantly differ from each other as it comes to education, and that BzRA use among the categories of work status significantly differ from each other as it comes to education.

The Poisson regression results in **model 1**_**a**_ (see Table 3) confirm a significant association between education and the use of BzRAs. Specifically, individuals with shorter (IRR 2.15, *P* < 0.001) and intermediate education (IRR 1.63, *P* < 0.001) report higher use rates than those with longer education. These educational differences remain significant in **model 1**_**b**_, which includes work status as a covariate. However, the Z-scores of the different education categories decline, suggesting a mediating effect of work status (Z-scores of model 1_a_ compared to model 1_b_: shorter education = 5.56/3.60, intermediate education = 4.71/2.97). In addition, work status is significantly related to BzRA use, irrespective of education. Specifically, unemployed (IRR 1.89, *P* < 0.001) and (pre-)retired individuals (IRR 2.13, *P* < 0.001) use them significantly more than employed individuals. There is no significant difference in use between non-employed (IRR 1.28, *P* = 0.128) and employed individuals. Sick or disabled individuals (IRR 5.83, *P* < 0.001) use them significantly more than employed individuals, which is not surprising given their health status.

**Table 3:** Weighted IRRs, Z-scores, and corresponding *P*-values of BzRA use in the past 24 h according to education, work status and the covariates.

	**Model 1**		**Model 2**	
	**Model 1**_a_	**Model 1**_b_	**Model 2**_a_	**Model 2**_b_
	**IRR (Z-score)** ***P*****-values**	**IRR (Z-score)** ***P*****-values**	**IRR (Z-score)** ***P*****-values**	**IRR (Z-score)** ***P*****-values**
**Education (ref.cat.: longer education)**				
Intermediate education	1.63 (4.71)^***^	1.37 (2.97)^***^	1.47 (3.78)^***^	1.31 (2.58)^*^
Shorter education	2.15 (5.56)^***^	1.63 (3.60)^***^	1.75 (4.25)^***^	1.49 (3.06)^**^
**Work status (ref.cat.: employed)**				
Unemployed		1.89 (4.47)^***^		1.61 (3.41)^**^
Non-employed		1.28 (1.52)		1.22 (1.22)
Sick or disabled		5.83 (15.13)^***^		3.47 (9.96)^***^
(Pre-)retirement		2.13 (5.90)^***^		2.08 (5.91)^***^
**Household income (ref.cat.: high)**				
Mediate income	1.28 (1.98)^*^	1.09 (0.66)^*^	1.17 (1.29)	1.03 (0.24)
Low income	1.76 (4.60)^***^	1.21 (1.43)^***^	1.48 (3.20)^**^	1.13 (0.95)
(Missings)	1.05 (0.32)	0.98 (−0.14)	1.04 (0.28)	0.97 (−0.22)
Gender (ref.cat.: man)	1.63 (5.89)^***^	1.61 (5.73)^***^	1.35 (3.63)^***^	1.40 (4.01)^***^
**Age (ref.cat.: 18–34 year)**				
35–49 year	3.44 (6.59)^***^	3.10 (6.06)^***^	3.13 (6.19)^***^	2.91 (5.80)^***^
50–65 year	6.28 (10.05)^***^	4.52 (8.03)^***^	6.11 (10.03)^***^	4.67 (8.31)^***^
Birth country (ref.cat.: Belgian)	0.68 (−2.77)^**^	0.70 (−2.70)^**^	0.69 (−2.80)^**^	0.71 (−2.54)^*^
**Urbanization (ref.cat.: cities-agglomerates)**				
Suburban-urban	1.09 (0.83)	1.08 (0.76)	1.09 (0.93)	1.10 (1.02)
Rural	0.96 (−0.37)	1.01 (0.08)	0.98 (−0.16)	1.04 (0.33)
**Region (ref.cat.: Flanders)**				
Brussels	1.85 (4.62)^***^	1.82 (4.71)^***^	1.57 (3.39)^**^	1.59 (3.59)^***^
Wallonia	2.06 (8.11)^***^	2.01 (8.11)^***^	1.73 (6.40)^***^	1.74 (6.61)^***^
**Wave (ref.cat.: 2004)**				
2008	1.01 (0.05)	1.00 (−0.00)	0.96 (−0.37)	0.95 (−0.54)
2013	0.87 (−1.25)	0.82 (−1.83)	0.70 (−3.34)^**^	0.69 (−3.50)^***^
2018	0.71 (−3.05)^**^	0.65 (−4.01)^***^	0.59 (−4.95)^***^	0.55 (−5.49)^***^
**Household composition (ref.cat.: single)**				
Couple	0.65 (−5.04)^***^	0.76 (−3.23)^***^	0.78 (−3.18)^**^	0.82 (−2.29)^*^
(Other composition)	0.73 (−2.19)^*^	0.77 (−1.91)	0.82 (−1.44)	0.81 (−1.57)
Social contact (ref.cat: less than once a week)	0.59 (−5.05)^***^	0.71 (−3.31)^**^	0.80 (−2.29)^*^	0.86 (−1.51)
GP contact past 12 months (ref.cat.: yes)	4.17 (8.01)^***^	3.56 (7.21)^***^	3.35 (6.81)^***^	3.07 (6.47)^***^
Regular GP (ref.cat.: yes)	2.06 (2.58)^*^	1.86 (2.24)^*^	1.80 (2.10)^*^	1.72 (1.97)^*^
**Mental health variables**				
Depression complaints (ref.cat.: no)			1.81 (4.53)^***^	1.54 (3.28)^**^
Anxiety complaints (ref.cat.: no)			2.31 (6.60)^***^	2.04 (5.59)^***^
Sleeping problems			1.32 (5.93)^***^	1.31 (5.71)^***^
Intercept	0.00 (−16.08)^***^	0.00 (−15.86)^***^	0.00 (−17.16)^***^	0.00 (−16.84)^***^

IRR, Interval Rate Ratio. ^*^P-value < 0.05. ^**^P-value < 0.01. ^***^P-value < 0.001.

In **model 2**_**a**_, which includes the mental health variables, education remains significantly associated with BzRA use, with the same interpretation as in **model 1**_**a**_. In **model 2**_**b**_, where work status is included, educational differences remain significant, but the Z-scores indicate a decline in the strength of the associations between the education categories and BzRA use (Z-scores of model 2_a_ compared to model 2_b_: shorter education = 4.25/3.06, intermediate education = 3.78/2.58). This again suggests that the observed educational differences in use are partially related to differences in work status, and independent from mental health status. Furthermore, significant differences in use according to work status remain, suggesting that the impact of work status on BzRA use is subject to medicalisation processes.

Regarding the covariates, it is noteworthy that there is a significant income gradient in BzRA use, with individuals from households with a lower income having higher use rates than those from households with a higher income. However, differences in mental health status explain this gradient completely, as evidenced by model 2_b_. In addition, men, non-Belgians and younger working-age adults report lower levels of BzRA use. Conversely, individuals living in Brussels or Wallonia tend to use them more than those residing in Flanders. Moreover, single people and those with infrequent social contact have higher use rates compared to those in relationships or with more frequent social contact. Having a regular GP and recent GP contact are also associated with higher use rates. In 2018, the most recent wave, BzRA use was significantly lower than in 2004 in Model 1_a_ and Model 1_b_. In Model 2_a_ and Model 2_b_, this trend already started from 2013 onwards. Additionally, depression-, anxiety- and sleeping complaints are strongly associated with higher use rates.

**Figure 1:**
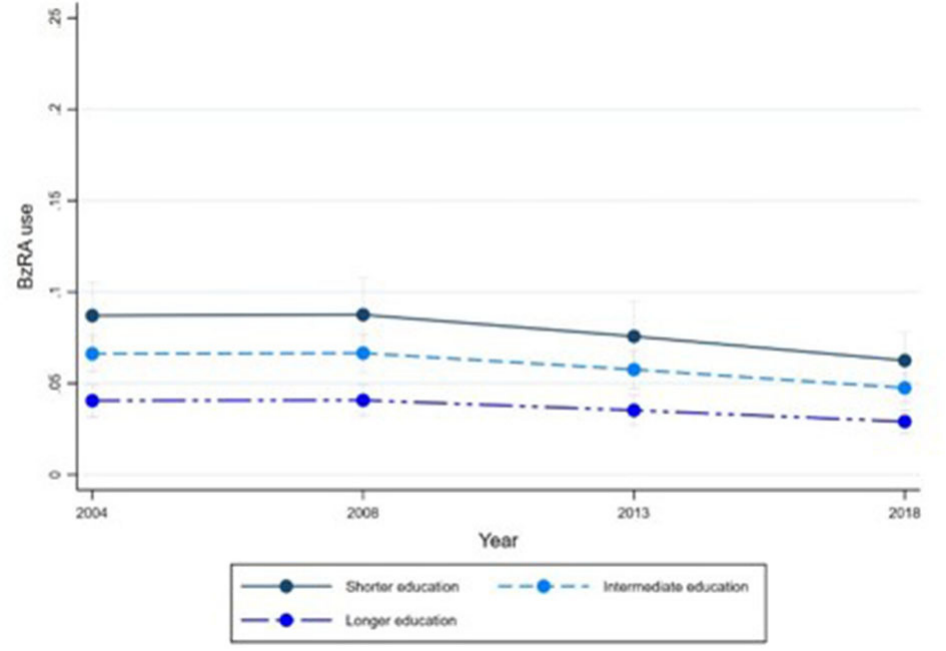
BzRA use in the past 24 h according to education and work status, stratified by wave, weighted prevalence (min.-max.:0–1) for Model 1 (see Table 3). Model 1_a_ (Education, before including work status).

**Figure 2:**
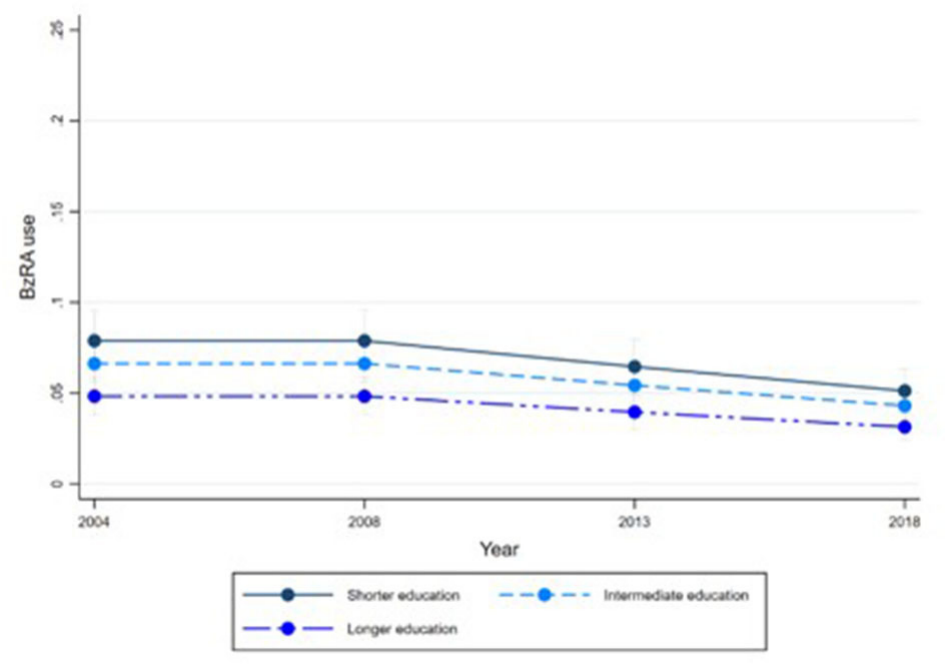
BzRA use in the past 24 h according to education and work status, stratified by wave, weighted prevalence (min.-max.:0–1) for Model 1 (see Table 3). Model 1_b_ (Education, after including work status).

**Figure 3:**
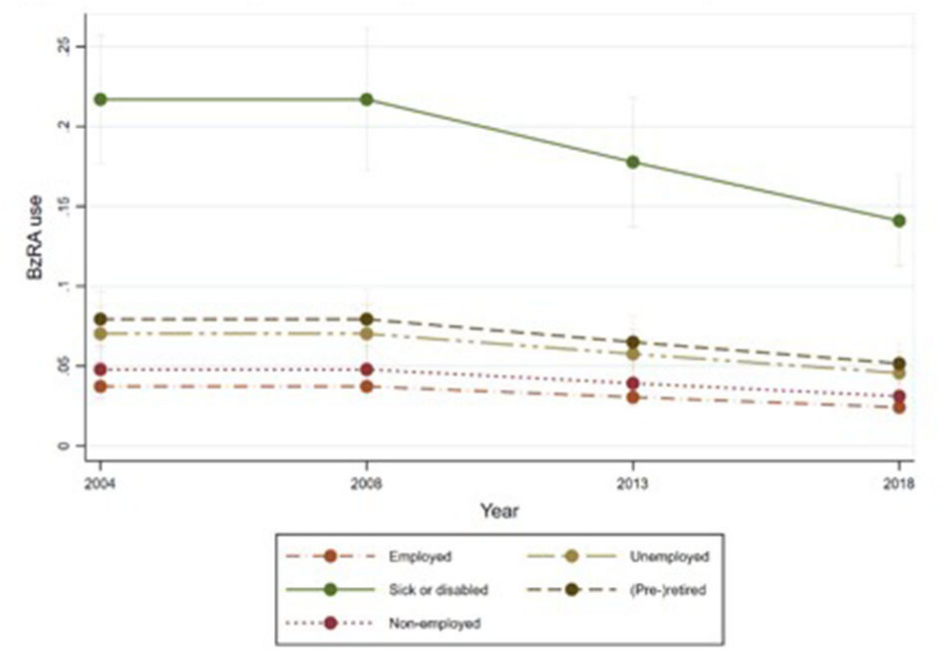
BzRA use in the past 24 h according to education and work status, stratified by wave, weighted prevalence (min.-max.:0–1) for Model 1 (see Table 3). Model 1_b_ (Work status, controlled for education).

**Figure 4:**
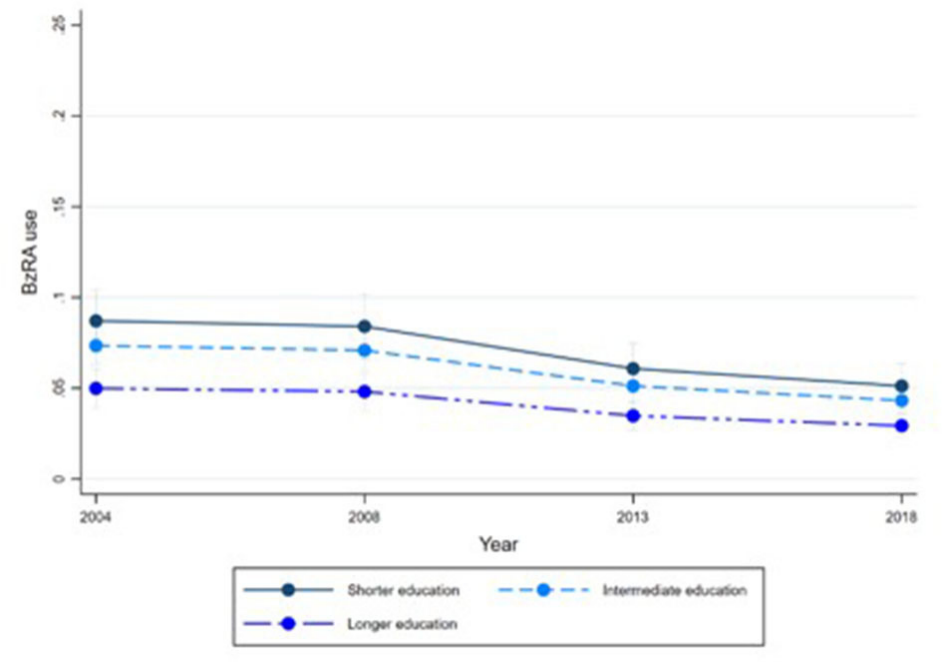
BzRA use in the past 24 h according to education and work status, stratified by wave, weighted prevalence (min.-max.:0–1) for Model 2 (see Table 3). Model 2_a_ (Education, before including work status).

**Figure 5:**
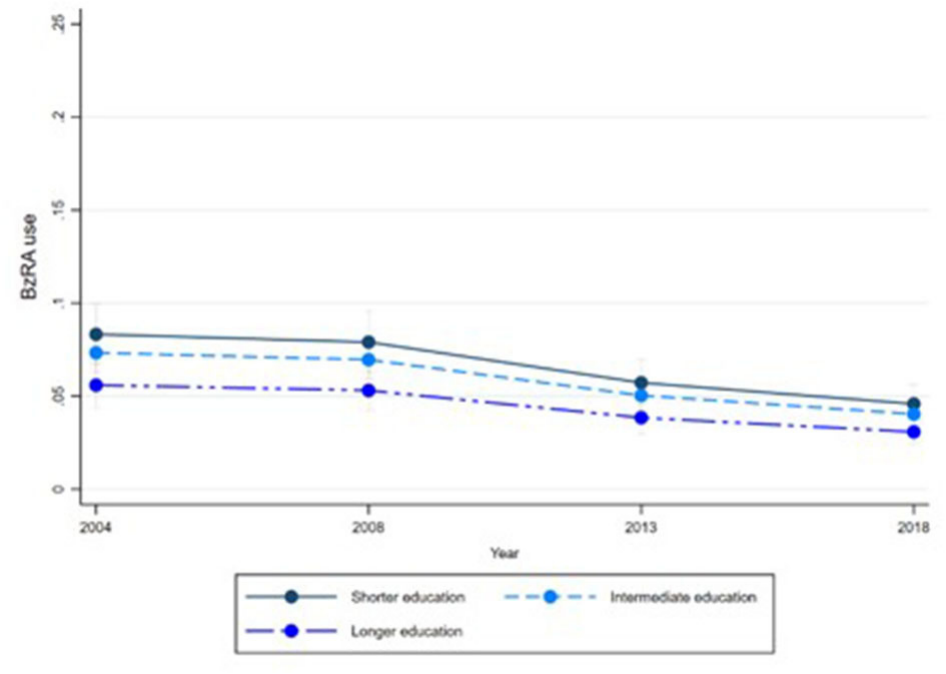
BzRA use in the past 24 h according to education and work status, stratified by wave, weighted prevalence (min.-max.:0–1) for Model 2 (see Table 3). Model 2_b_ (Education, after including work status).

**Figure 6:**
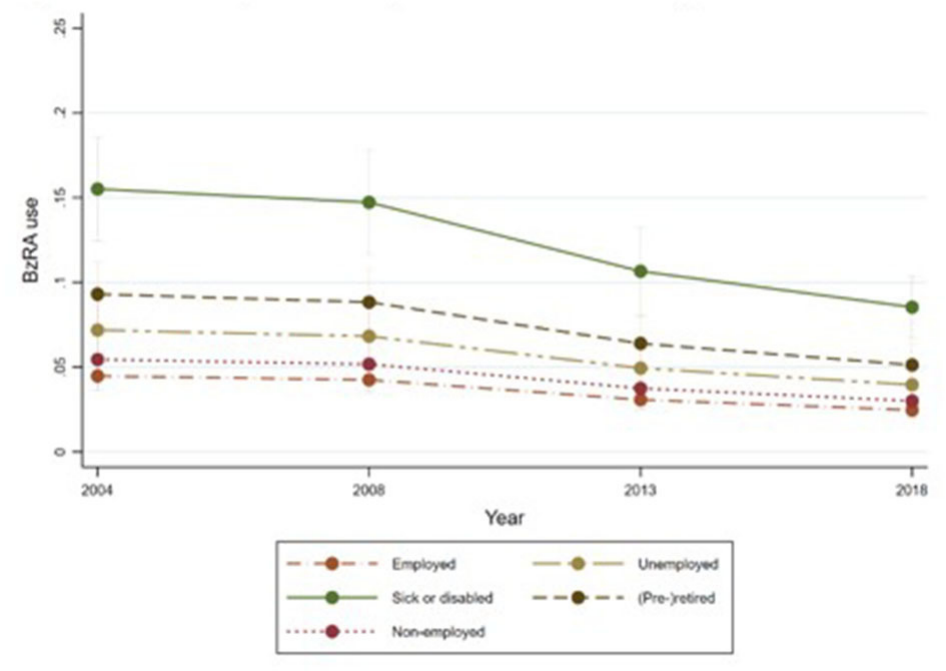
BzRA use in the past 24 h according to education and work status, stratified by wave, weighted prevalence (min.-max.:0–1) for Model 2 (see Table 3). Model 2_b_ (Work status, controlled for education).

Figures 1–6 display Poisson regression graphs depicting trends over time. The graphs reveal that the addition of work status (model 1_a_ and model 2_a_ compared to model 1_b_ and model 2_b_) reduces educational differences, suggesting that work status mediates this relationship. When comparing model 2_a&b_ to model 1_a&b_, inclusion of the mental health variables results in decreased inequalities and highlights the general declining trend in BzRA use. However, the graph of model 2_b_ still displays differences in use based on individuals' work status, regardless of their education or mental health status.

## Discussion

Our study reveals several important findings. Firstly, work status acts as a mediator, partially explaining educational differences in BzRA use, even after accounting for mental health status. We found that individuals who are unemployed, (pre-)retired, or sick or disabled, regardless of their mental health status, exhibit higher levels of use than employed individuals. This aligns with previous research, which suggests that work-related uncertainty may lead to increased prescribing and medication use, regardless of mental health (36–39). Medicalisation and pharmaceuticalisation processes tend to detach social problems from their social roots and treat them as personal failures (40, 41). The marginalization of the social roots of unemployment, sick leave and pre-retirement has led to a personalization of responsibility. Negative feelings, including stress and self-stigma, arising from such work statuses may cause isolated, non-specific symptoms for which medical treatment is sought, including psychotropics like BzRAs (28).

Secondly, we found that even after taking into account mental health status, part of the educational differences in BzRA use remain unexplained, as work status only partially mediates the relationship. This finding is consistent with a recent Belgian study, which highlights the role of education as a socialization mechanism in explaining differences in mental health care use, rather than allocation mechanisms (16). The study suggests that education plays a significant role in influencing mental health care use, as it enhances non-material resources such as knowledge and social competences that may impact health care use (17). Socialization mechanisms contribute to creating social inequalities, as individuals with longer education are more likely to choose ‘effective' treatment options (42, 43), or in the case of BzRAs, to neglect ‘non-effective' options, due to their awareness of potential side- and withdrawal effects. Moreover, longer educated individuals also tend to adopt a certain ‘health identity' (44).

Furthermore, we observe an overall declining trend in BzRA use, which is an important pattern, especially when the mental health variables are taken into account. This suggests that non-need-based use is decreasing over time, possibly due to numerous prevention campaigns and federal government initiatives aimed at raising awareness of the side- and withdrawal effects of these medications (7, 9, 45). However, despite this trend, overconsumption remains a significant problem, with a large group of individuals continuing to use them daily on a long-term basis (10, 46). Studies indicate that while GPs are aware of the issues of overconsumption and long-term use, they often struggle to support their patients in stopping their use (39, 47, 48). In fact, research suggests that GPs find it hard to take initiative to change their chronic prescribing behavior (39, 49). Therefore, although a declining trend is visible, future research should focus on (inequalities in) the prevalence and incidence of long-term BzRA use and how it evolves over time, as well as strategies to address their long-term use.

Our study also reveals some expected findings, such as the relationship between mental health complaints and BzRA use. Specifically, higher levels of complaints are associated with higher levels of use. However, it is important to note that the use of BzRAs does not necessarily eliminates all complaints, suggesting a sub-optimal relationship between use and mental health. This could be because they are not true ‘medicines' but rather medication that enable individuals to function socially despite complaints (50). In addition, our study finds a positive association between higher levels of BzRA use and more frequent GP visits, which is expected given that GPs often prescribe these medications (48). However, this association could also highlight the presence of a GP-medication nexus that is not necessarily connected to the level of patient's complaints or their social context. Instead, it may be a result of GPs being ‘locked' into a particular mode of treatment (51, 52).

Two limitations of this study also need to be addressed. The first limitation concerns the measurement of the dependent variable BzRA use. Respondents were asked to present the medicines they had used in the past 24 h, and the interviewer then matched these to appropriate ATC codes on a list. Although the interviewer verified the medicines, respondents may not have shown all the medicines they had used, especially for stigmatized medicines such as BzRAs (53, 54). Longer educated individuals may be particularly aware of the stigma surrounding psychotropic medication use, including the potential risks of use (55), which could affect their willingness to disclose BzRA use to the interviewer. Since the question was part of a general module on the use of medicines, unrelated to the issue of mental health, the potential impact of self-report bias may be limited. Nevertheless, the reported rates probably underestimate the consumption of BzRAs. Furthermore, obtaining more information about the medication use would have been beneficial, including whether BzRAs were used to treat specific symptoms or diagnoses and whether they were used infrequently or frequently. The second limitation concerns the lack of comprehensive work-related information in the BHIS. It would have been interesting to include more detailed information on working conditions and work arrangements, related to the psychosocial quality of labor, which are associated with both education (56) and mental health care use (57, 58).

To end, some findings of this study require further investigation. For example, the higher use of BzRAs among older working-age adults beyond their (mental) health status and social position. Future research may investigate how their higher use is related to their GP contact. It may be possible that the increased use is not solely due to the age or cohort of the users (59), but also to the age or cohort of the GPs who are prescribing the medications (60). This finding highlights the need to understand the factors contributing to the prescribing practices of GPs.

Another finding that deserves consideration is the lower level of BzRA use among non-Belgians. For individuals from neighboring countries, the lower use could be explained by the fact that GPs play a less central role in those countries, with the exception of France, resulting in lower use of psychotropics (61). In Belgium and France, there is a low rate of referral to mental health specialists and a low rate of mental health specialist consultations. In contrast, in the Netherlands there is a high rate of referral and non-medical mental health care use. For individuals from non-neighboring countries, this may be due to their underrepresentation in ambulatory health care, which could be caused by cultural-specific barriers such as language barriers or differences in preferences and health care seeking behavior (62–64). Barriers can also occur at the level of health care providers, where patients from non-neighboring countries are treated differently, which is described as provider bias (65, 66).

Lastly, our study also indicates regional disparities in BzRA use, with higher levels of use observed in Brussels and Wallonia, even after adjusting for socio-demographic and composition factors. In Belgium, the competences for health care, including mental health care and health promotion, are decentralized, with the federal government retaining only certain competences (67). This decentralization, combined with the multiple communities with different traditions and behavioral patterns that make up Belgium, might contribute to the regional differences in use. However, future research is needed to gain a more comprehensive understanding of the factors driving these regional differences.

## Conclusion

In conclusion, work status plays a crucial role in explaining educational differences in BzRA use, regardless of mental health status. This indicates that non-medical factors, such as work status, are medicalized and associated with medical mental health care-seeking behavior. Health care providers should be cautious while prescribing BzRAs and consider non-medical factors that may influence a patient's decision to seek medical treatment. Given the potential risks of BzRA use and the possibility of non-medical underlying reasons, health care providers should prioritize non-pharmacological treatment options. Therefore, it is essential for health care providers to educate their patients on the potential risks of BzRA use and the importance of non-pharmacological therapies.

## Data availability statement

The data analyzed in this study is subject to the following licenses/restrictions: the data will not be deposited since its subject to a contract with Sciensano (The Scientific Institute of public health of the federal Belgian State). Requests to access these datasets should be directed to https://www.sciensano.be/en/node/55737/health-interview-survey-microdata-request-procedure.

## Ethics statement

Ethical review and approval was not required for the study on human participants in accordance with the local legislation and institutional requirements. Written informed consent for participation was not required for this study in accordance with the national legislation and the institutional requirements.

## Author contributions

LC designed the study, had primary responsibility for the writing and editing of the manuscript, and performed the data analysis. KD, PB, and MC critically reviewed the manuscript. All authors gave final approval for the article to be published.

## Footnotes

^1^Following ATC level four classes were included: N03AE, N04BA, N05CD and N05CF.
