# Supplementary material for: Trends in the use of benzodiazepine receptor agonists among working-age adults in Belgium from 2004 to 2018
Source: Front Public Health. 2023 Jun 15;11:1191151. doi: 10.3389/fpubh.2023.1191151 (PMC10311493; doi:10.3389/fpubh.2023.1191151)
Supplement: Supplementary file 1 [file Data_Sheet_1.pdf]

Supplementary Material

Appendix 1a. Respondent selection criteria.

The initial merged sample size of the BHIS, which consisted of six waves, included 73,681 respondents. After excluding wave 1997 and 2001, because of lack of data on several important variables (N=23,556), and respondents not aged between 18 and 65 years old (N=20,639), 29,486 respondents remained. After further excluding missing values on the dependent variable and independent variables (N=10,939), the present study holds data from 18547 respondents.

Appendix 1b. Table with general sample characteristics.

	N	%		N	%
**Wave**			**Birth country**		
2004	5,045	27.20	Belgian	15,230	82.12
2008	4,412	23.79	Non-Belgian	3,317	17.88
2013	4,076	21.98	**Region**		
2018	5,014	27.03	Flanders	7,357	39.67
**Education**			Brussels	4,159	22.42
Longer education	1,427	7.69	Wallonia	7,031	37.91
Intermediate education	9,379	50.57	**Household composition**		
Shorter education	7,741	41.74	Single	4,987	26.89
**Work status**			Couple	11,997	64.68
Employed	13,317	71.80	Other composition	1,563	8.43
Unemployed	1,544	8.32	**Social contact**		
Non-employed	923	4.98	Less than once a week	1,958	10.56
Sick or disabled	1,661	8.96	More than once a week	16,589	89.44
(Pre-)retirement	1,102	5.94	**GP contact**		
**Household income**			Yes	14,629	78.88
High income	4,459	24.04	No	3,918	21.12
Mediate income	6,600	35.59	**Regular GP**		
Low income	4,951	26.69	Yes	17,331	93.44
(Missings)	2,537	13.68	No	1,216	6.56
**Gender (ref.cat.: man)**			**Depression complaints**		
Man	8,873	47.84	Yes	2,025	10.92
Woman	9,674	52.16	No	16,522	89.08
**Age**			**Anxiety complaints**		
18-34 year	4,732	25.51	Yes	1,693	9.13
35-49 year	6,812	36.73	No	16,854	90.87
50-65 year	7,003	37.76	**Sleeping problems**		
**Urbanisation**			Not at all	6,452	34.79
Cities-agglomerates	7,928	42.75	No more than usual	7,925	42.73
Suburban-urban	7,268	39.19	Rather more than usual	3,266	17.61
Rural	3,351	18.07	Much more than usual	904	4.87

Note. In the total sample, including four waves (2004-2018) N=18,547.

Appendix 2a. Operationalization of depression complaints.

Depression is measured using the PHQ-9 (34), a nine-item instrument that is used to assess the severity of depression complaints. The PHQ-9 consists of nine questions using the following wording: "*Over the last two weeks, how often have*

you been bothered by any of the following problems: (1) little interest or pleasure in doing things, (2) feeling down, depressed, or hopeless, (3) trouble falling or staying asleep, or sleeping too much, (4) feeling tired or having little energy, (5) poor appetite or overeating, (6) feeling bad about yourself - or that you are a failure or have let yourself or your family down, (7) trouble concentrating on things, such as reading the newspaper or watching television, (8) moving or speaking so slowly that other people could have noticed - or so fidgety or restless that you have been moving a lot more than usual, (9) thoughts that you would be better off dead, or thoughts of hurting yourself in some way" with four answer categories (not at all=0, several days=1, more than half the days=2, nearly every day=3). If more than two items have missing values, the indicator is set as missing. If less than 3 items have missing values, these are replaced by the mean score calculated from the remaining valid items on the subscale. In the original coding a distinction is made between major depression and other depression. Major depression is considered when five or more of the symptoms are present 'more than half the days (the 9th item counts if endorsed 'several days') and when one of the first two symptoms is endorsed. Other depression is considered when one of the first two symptoms are present (>0), and when two to four symptoms are present 'more than half the days' (the 9th item counts if endorsed at all (>0)). If major depression or other depression=1, depressive complaints=1; if major depression and other depression=0, depressive complaints=0.

Appendix 2$_b$. Operationalization of anxiety complaints.

Anxiety is measured using the GAD-7 (35), a seven-item instrument that is used to assess the severity of anxiety complaints. The GAD-7 consists of seven questions using the following wording: "*Over the last two weeks, how often have you been bothered by any of the following problems: (1) feeling nervous, anxious, or on edge, (2) not being able to stop or control worrying, (3) worrying too much about different things, (4) trouble relaxing, (5) being so restless that it's hard to sit still, (6) becoming easily annoyed or irritable, (7) feeling afraid as if something awful might happen"* with four answer categories (not at all=0, several days=1, more than half the days=2, nearly every day=3). If more than two items have missing values, the indicator is set as missing. If less than 3 items have missing values, these are replaced by the mean score calculated from the remaining valid items on the subscale. The summed item values are dichotomized using 10 as a cut-off score, with scores higher than 10 signaling moderate to severe levels of anxiety.
